# Proactive Geriatric Medication Management and Deprescribing Efforts in Swiss Nursing Home Residents

**DOI:** 10.3390/jcm14072142

**Published:** 2025-03-21

**Authors:** Julian Gsell, Sandro Baumgartner, Mathias Schlögl, Katrin Leenen, Markus Béchir, Stefan Russmann

**Affiliations:** 1 Department of Chemistry and Applied Biosciences, Swiss Federal Institute of Technology Zurich (ETHZ), 8093 Zurich, Switzerland; julian.gsell@hotmail.com; 2 Centre for Internal Medicine, Clinic Hirslanden Aarau, 5001 Aarau, Switzerlandmarkus.bechir@zim.ch (M.B.); 3 Department of Geriatric Care, Clinic Barmelweid, 5017 Barmelweid, Switzerland; 4 Private Psychiatric Practice Dr. Leenen, 5000 Aarau, Switzerland; 5 Faculty of Medicine, University of Nicosia, 2408 Engomi, Cyprus; 6 Clinical Pharmacology, Pharmacoepidemiology and Pharmacogenetics, Drugsafety.ch, 8703 Küsnacht, Switzerland

**Keywords:** polypharmacy, geriatrics, deprescribing, clinical decision support, pharmavista

## Abstract

**Background/Objectives**: Polymorbidity and polypharmacy are major challenges in geriatric care, resulting in a reduced quality of life and increased health care costs. **Methods**: We evaluated the proactive medication management of nursing home residents through personal visits and the use of a clinical decision support system (CDSS) with an integrated Beers Criteria list. **Results**: Among 56 nursing home residents, we observed a high prevalence of polypharmacy with an average of 7.9 regular and 5.1 on-demand prescriptions. Proactive medication management led to persistent medication changes in 87.5% of patients. Regular prescriptions were reduced in 21 residents and increased in 18 residents, resulting in a reduced use of cardiovascular drugs and antacids (*p* < 0.05), but no significant overall reduction in polypharmacy. CDSS alerts based on Beers Criteria made no clinically relevant contribution to medication reduction. **Conclusions**: Proactive geriatric medication management led to persistent medication changes and no reduction in overall polypharmacy but reduced the use of selected drug classes that are associated with an increased risk of adverse reactions and costs. The clinical relevance and implementability of Beers Criteria were low, revealing major limitations of algorithm-based alerts for older patients, who require additional personalized evaluations of their individual complex healthcare needs.

## 1. Introduction

Polypharmacy is the simultaneous use of multiple medications (usually five or more) in patients with multiple medical conditions (polymorbidity) and may also occur due to system-level factors such as continued medical therapy without the original indication (failure to deprescribe). Polypharmacy is associated with an increased risk of drug–drug and drug–disease interactions, both of which can lead to adverse drug events (ADEs) that increase morbidity, mortality, and healthcare costs [1,2,3,4].

Switzerland has reportedly the lowest prevalence of polypharmacy in older people in Europe, but at 26.3%, it is still high [5], and it is even much higher in nursing home residents. Nationwide, 42.6% of nursing home residents take nine or more different substances daily [6]. Of note, nursing home residents taking ≥9 different medications were reported to be 2.3 times more likely to experience at least one ADE [7].

Due to the demographic population change with an increasing proportion of older people, polypharmacy in geriatric care is a growing concern. To address this problem, medication lists should be actively monitored, and medications without a clear indication and no beneficial effect should be deprescribed. When deprescribing, clinicians should consider the patients’ and the health professionals’ perspectives on the goals of therapy, including disease progression, the prevention of health decline, symptom management, and personal preferences [8]. On the other hand, even in older patients with pronounced polypharmacy, there may be a justified need for additional prescriptions, particularly in palliative situations.

Clinical decision support systems (CDSSs) can help physicians identify potential medication errors, and pharmavista is a CDSS that also includes an automated check of the Beers Criteria list of potentially inappropriate medications (PIMs) in adults over 65 years of age [9]. Due to their high sensitivity, CDSSs are suitable tools to screen for potential medication errors and unnecessary polypharmacy. However, an automated CDSS check lacks information on the history of drug effects in an individual person, or the person’s personal perspective on the goals of therapy. This contributes to their low specificity when used in clinical practice, i.e., most automated CDSS alerts are clinically irrelevant, and irrelevant alerts consequently result in poor acceptance and indiscriminate overriding [10,11]. This limitation may also apply to drug–age interaction alerts.

Therefore, the aim of this study was to describe and evaluate the outcome of proactive medication management in nursing home residents, with a focus on the deprescribing and optimization of medication, and to determine the clinical value of drug–age interaction alerts from CDSSs.

## 2. Materials and Methods

### 2.1. Study Design, Setting, Patient Population, and Procedures

This observational study analyzed proactive geriatric medication management using an interdisciplinary team of physicians in residents of four Swiss nursing homes between May 2023 and June 2024. These four nursing homes are all located at the center of a Swiss city with approximately 23 thousand inhabitants. Previously, general practitioners were responsible for the basic medical care of the nursing home residents, but changes in the health care system made it increasingly difficult for general practitioners to provide such a service. Therefore, the department of general internal medicine and emergency services of a nearby tertiary care hospital offered to provide medical care for all nursing home residents with their interdisciplinary team of internists, geriatricians, psychiatrists, and clinical pharmacologists. This team also introduced a rotation system of weekly visits in one of the four nursing homes and systematic proactive medication management.

First, all prescriptions of nursing home residents were screened for potential medication errors using the CDSS pharmavista (see Section 2.2 below), and all resulting CDSS alerts were assessed by a medical student (J.G.) and a senior clinical pharmacologist (S.R.) for clinical relevance in the context of all available medical information for each individual resident. This involved reviewing all relevant information from electronic medical records (EMRs), including diagnoses, laboratory results, current conditions, and treatment plans. During the EMR review, we specifically assessed each resident’s medication plan for potential drug–drug, drug–age, drug–disease, and drug–food interactions, as well as the dose, therapeutic duplications, and other potential risk factors for ADEs, such as renal impairment, liver disease, or abnormal laboratory results. The methods used to identify medication errors and factors of clinical relevance were identical to those used in a previous study published by our group [10]. Special attention was paid to the possibility of deprescribing, particularly for benzodiazepines/Z-drugs, antipsychotics, and antidepressants. Furthermore, we also evaluated each resident regarding potentially new indicated prescriptions. Following EMR-based evaluations of the medication for each resident, we provided a written clinical pharmacological report with recommendations for adjustments to the current medication and monitoring. Recommendations were subsequently reevaluated by specialists in internal medicine and geriatric care or the clinical pharmacologist during personal nursing home visits under consideration of the residents’ individual current needs, and recommendations were adjusted and implemented as appropriate. Upon a long-term follow-up check after several months, we then documented and compared the pharmacotherapy of the nursing home residents before and after proactive medication management to assess the lasting effects of our adjustments.

### 2.2. Clinical Decision Support Systems (CDSSs)

Screening for potential medication errors was performed using the CDSS pharmavista (https://pharmavista.ch) with manual entry of all patient data and prescriptions. We documented all automated alerts generated by the CDSS for each resident. Pharmavista provides the output of its automated medication check-in tables stratified over the following categories: drug–drug interactions, therapeutic duplications, excess of maximum recommended dose, drug–age interactions according to the Beers Criteria list [9] and summary of product characteristics (SPC), and drug–food interactions. If a resident’s estimated glomerular filtration rate (eGFR) was available, we also used pharmavista’s feature to check for recommended dose adjustments in response to impaired renal function.

### 2.3. Outcomes and Data Analysis

The primary outcomes of this study were a quantitative and qualitative presentation of CDSS-generated medication alerts in geriatric nursing home residents and a comparison of these to the actual implementation of CDSS alerts and other lasting changes in the prescribed medication by senior physicians.

Data analysis was descriptive, with the results being presented in tables and figures. Averages were expressed as mean or median and range, as appropriate. Renal function was categorized based on the estimated glomerular filtration rate (eGFR) calculated with the CKD-EPI formula. Medication changes measured as the mean number of medications per patient after proactive medication management vs. baseline were compared using a two-sided *t*-test.

We used Microsoft Excel 16.95.1 for MacOS (Redmond, Waukesha, WI, USA) and STATA 17.0 for MacOS (STATA Corp., College Station, TX, USA) for data management, descriptive statistics, and the design of the graphical presentation of the results.

## 3. Results

### 3.1. Characteristics of Nursing Home Residents

We screened 68 nursing home residents above the age of 65 years with at least one regular prescription per day regarding their current medication and all available medical information. Among those, ten were excluded from the analysis due to death occurring between medication review and implementation, and two were discharged from the nursing home before implementation and follow-up. The remaining 56 residents were included in the final analysis. Their demographics and most recent laboratory results for serum sodium, potassium, and eGFR are shown in Table 1. The median age was 91 years, with a range from 74 to 100 years; there was a predominance of women (75%); and all residents were White. At baseline, renal function was impaired with an eGFR between 30 and 59 mL/min/1.73 m^2^ in 29.2% of residents, and below 30 mL/min/1.73 m^2^ in 8.3%. Hypokalemia was observed in 10.7%, and hypo- or hypernatremia in 5.4% and 1.8%, respectively.

### 3.2. Drug Prescriptions

**Figure 1:**
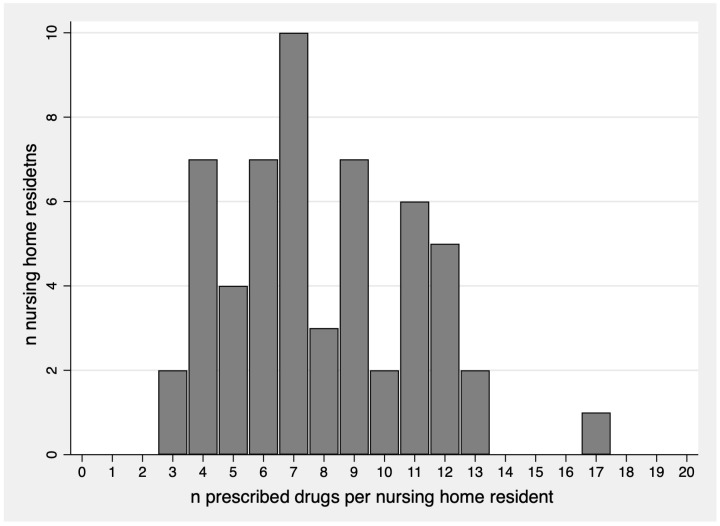
Distribution of polypharmacy in 56 nursing home residents.

Figure 1 and Table 2 present an overview of drug prescriptions in the study population. Nursing home residents had a mean of 7.9 (range 3–17) regular daily prescriptions and 5.1 (range 0–13) on-demand prescriptions. The proportion of residents regularly taking five or more different substances per day was 76.8%, and for nine or more, it was 41.1%. The four most prescribed drug classes were antihypertensives/diuretics, prescribed in 73.2% of residents, followed by anticoagulants/antiplatelets (53.6%), antiarrhythmics (including beta blockers, which we classified as antiarrhythmics, but they also have antihypertensive effects), and proton pump inhibitors (both 44.6%). Antipsychotics, antidepressants, and benzodiazepines/Z-drugs were regularly prescribed in 42.9%, 37.5%, and 21.4% of patients, respectively.

### 3.3. Frequency of CDSS Alerts for Potential Medication Errors

CDSS alerts are summarized in Table 3. On average, pharmavista generated 12.7 alerts per resident (range 1 to 35). The most severe alerts (level 1) were rare with a mean of 0.3 alerts per resident, affecting 20% of our residents. In contrast, level 2 and 3 alerts were very common with a mean of 5.7 and 6.7 alerts per resident, respectively. Drug–drug interaction alerts accounted for 44% of all alerts, and renal impairment alerts accounted for for 12%. For drug–age interactions, pharmavista implemented the Beers Criteria list and the summary of product characteristics (SPC). For our medication checks, we had pharmavista’s default filter for drug–age interactions activated, and with this default setting, pharmavista generated, on average, 0.9 age-related alerts per resident from the Beers Criteria list, 83% of which were classified as level 2. When we deactivated the default filter for Beers alerts, pharmavista displayed 4.8 alerts overall per resident based on Beers Criteria, i.e., more than 5 times more alerts than with the activated filter.

### 3.4. Medication Adjustments After Systematic Evaluation

Following the systematic evaluation of the medication, we adjusted prescribing and monitoring upon personal visits of nursing home residents. A follow-up check was performed after a median time of 307 days (after the initial evaluation of medication), and lasting medication adjustments were documented in 49 out of 56 residents (87.5%).

**Figure 2:**
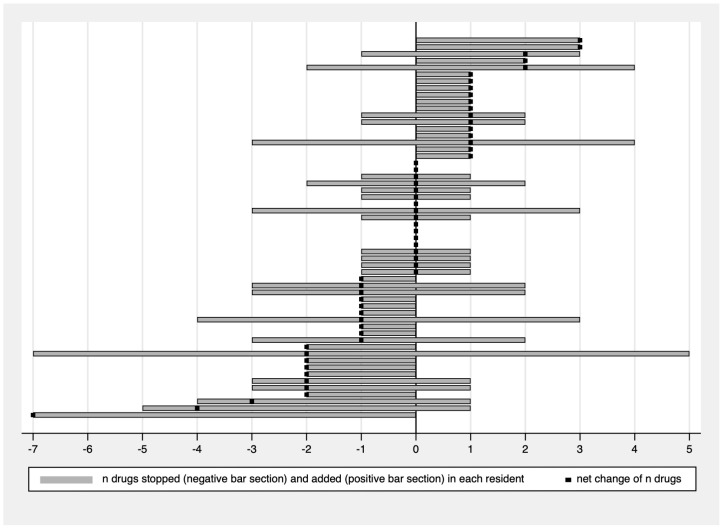
Deprescribing and additional prescribing in each individual resident following proactive medication management (regular prescriptions only).

Figure 2 presents medication changes in regular prescriptions by showing the number of drugs added (positive section of each bar on the right), the number of drugs stopped (negative section of each bar on the left), and the resulting net change in the total number of prescribed medications (black squares) for each of the 56 individual nursing home residents. As shown, there were, for example, only seven residents (12.5%) where we neither added nor withdrew a prescription (residents without bars and net change zero), two residents had three additional prescriptions but no deprescribing (top of Figure 2), and in one resident, we were able to deprescribe seven medications without adding any other (bottom of Figure 2). Figure 2 also shows that, at the time of follow-up, there was a net deprescribing in 21 residents (37.5%), net additional prescribing in 18 residents (32.1%), and no net change in the total number of regular prescriptions in 17 residents (30.4%). Looking separately at reduced and additional prescriptions, there were 36 residents (64.3%) with at least one stopped regular prescription and 37 residents (66.1%) with at least one additional regular prescription.

On average, 1.4 drugs per resident were withdrawn and 1.1 drugs were added, resulting in no significant decrease in overall polypharmacy with a mean number of 7.9 regular prescriptions per resident before vs. 7.7 after proactive medication management (*p* > 0.05).

For on-demand prescriptions, the mean number of medications increased, i.e., from a mean value of 5.1 per resident before to 6.8 after proactive medication management (*p* < 0.05), which reflects our deprescribing efforts by changing regular prescriptions to on-demand.

### 3.5. Medication Changes Stratified by Medication Class

**Figure 3:**
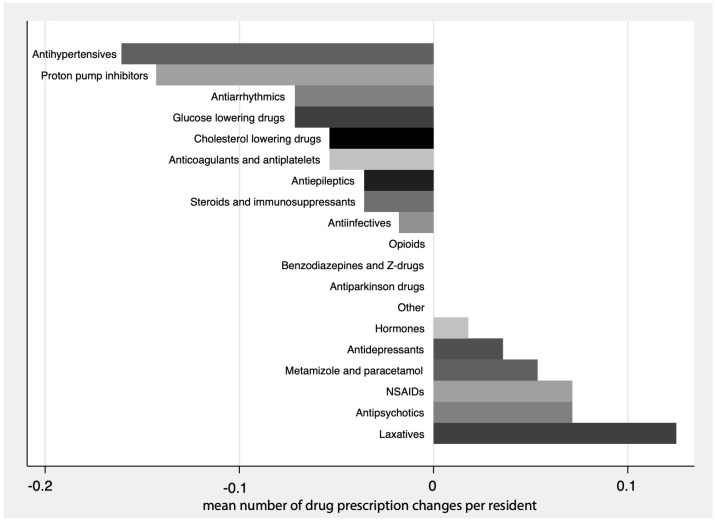
Mean number of changes per resident of regular prescriptions by medication class following proactive medication management (regular prescriptions only).

Figure 3 presents the average changes in regular prescriptions for all 56 residents with stratification according to different medication classes. As shown, medication classes that could be reduced overall (top section of Figure 3) were antihypertensives and diuretics, proton pump inhibitors, antiarrhythmics (which include beta blockers in the classification we used and therefore a further reduction in drugs with antihypertensive properties), glucose-lowering drugs (mainly metformin in response to impaired renal function with an eGFR < 30 mL/min/1.73 m^2^), cholesterol-lowering drugs, and low-dose aspirin (when initially prescribed for the primary prevention of ischemic vascular events, but no indication according to more recent evidence). These changes were statistically significant for antihypertensives and diuretics (mean delta number of drugs per resident at follow-up after proactive medication management vs. baseline = −0.16, *p* < 0.05) and for proton pump inhibitors (−0.14, *p* < 0.05). For other medication classes, there was a trend towards a small non-significant increase rather than decrease in prescriptions following our clinical evaluation and medication management, including laxatives, antipsychotics, and non-opioid analgesics.

Of further note, we stopped prescriptions for thick liquid paraffin laxatives in residents with Parkinson’s disease and other causes of swallowing disorders because of the risk of lipoid pneumonia, and we replaced them with safer alternatives such as macrogol.

Some drugs acting on the central nervous system (CNS), namely antipsychotics, antidepressants, and benzodiazepines or Z-drugs, have a particular reputation for being overprescribed in older people because they may have limited benefits and instead cause avoidable adverse drug reactions. As shown in Table 3, 49.1% of level 2 CDSS alerts are based on the Beers Criteria list, and CNS drugs account for a major part of these alerts. Therefore, we had a special focus on CNS drugs for proactive medication management, including documentation of within-class polypharmacy and dose adjustments. As shown above (Table 2), regular prescriptions of antidepressants, antipsychotics, and benzodiazepines/Z-drugs were indeed frequent in our nursing home residents. However, the clinical significance of alerts related to CNS drugs was low in the individual clinical context of our residents, leading to only minor non-significant changes in prescriptions for antipsychotics (+0.07, *p* > 0.05), antidepressants (+0.04, *p* > 0.05), and benzodiazepines or Z-drugs (±0) following our clinical evaluation.

## 4. Discussion

This study evaluated the outcome of proactive medication management in nursing home residents. We observed a high prevalence of polypharmacy, and proactive medication management with personal visits and systematic medication evaluation supported by CDSSs led to persistent prescription changes in 87.5% of residents. However, overall polypharmacy was not reduced, and the contribution of drug–age interaction alerts from CDSSs based on Beers Criteria was limited in clinical practice.

Most residents welcomed a systematic review of their medication through our on-site visits, as many complained about taking “too many pills a day”. Nursing staff also responded positively and contributed helpful suggestions on adjustments, particularly as they may have known the residents for a long time and could predict their needs and how they would respond to changes. Nevertheless, some residents are generally reluctant to make any changes in their medication, and others may experience temporary withdrawal symptoms upon stopping certain medications. To counteract this, we personally discussed planned medication changes with residents and aimed to gradually reduce rather than stop medications when appropriate. For example, sedatives, painkillers, or PPIs were often first reduced or switched to on-demand prescriptions. Accordingly, we allowed for a long period between medication changes and follow-up documentation to be sure that changes were persistent, whereas changes that were not tolerated and had to be switched back to the original medication would not be documented and evaluated as successful.

In our population, 41.1% of residents regularly took nine or more medications, which is closely similar to the proportion of 42.6% reported in a nationwide survey of nursing home residents [6] and therefore supports the validity and national representability of our results. Comprehensive evaluations with personalized decisions led to a dynamic mix of deprescribing, on the one hand, and additional prescriptions, on the other hand, resulting in persistent changes in prescriptions in 87.5% of nursing home residents. At the same time, it may be unexpected that our medication management did not result in an overall reduction in polypharmacy, as a reduction in polypharmacy was one of our declared aims. However, our deprescribing efforts are primarily aimed at low-benefit and high-risk medication groups. As such, antihypertensive drugs could often be reduced when recent blood pressure readings were low, but if high, antihypertensives even had to be intensified. This compares to the results of a recent study from the United States, where antihypertensives could be reduced in 16% and were intensified in 17% of long-term care residents [13]. Other studies reported that, among residents with dementia, deprescribing of antihypertensives was associated with 16% reduced odds of cognitive decline and a reduced risk of falls [14,15]. And a prospective Norwegian study with a tailored approach led to a reduction in polypharmacy that was mainly driven by withdrawals and dose reductions in cardiovascular drugs, followed by the improvement of a health-related quality of life score [16]. These reports support the assumption that our overall deprescribing of antihypertensives has positive long-term effects. Besides antihypertensives, proton pump inhibitors (PPIs) also had a high prevalence and could be reduced overall. This finding is in line with the results of a recent study on Australian nursing home residents [17], and deprescribing efforts of PPIs have also been shown to be cost-effective and save quality-adjusted life years (QALYs) [18].

The lack of an overall reduction in polypharmacy appears to contrast with the high number of CDSS alerts, particularly with the high number of alerts against PIMs in the elderly based on the Beers Criteria list that affected more than 50% of the residents in our population. However, the clinical relevance and implementability of these alerts appear limited. We have previously shown that CDSSs generally create considerable over-alerting and that, in clinical practice, they should be considered as screening tools with high sensitivity, although their specificity regarding clinical relevance is low [10,11]. Over-alerting appears to be even more pronounced for CDSS alerts based on Beers Criteria in older people. Two other recent studies from Sweden and Canada reported similar findings to ours, i.e., most alerts were clinically irrelevant or of low priority, and pharmacotherapy interventions led to only a small decrease in polypharmacy, but no decrease in PIMs based on Beers Criteria [19,20]. Furthermore, even though PIMs are associated with hospitalizations [21], they may just be a proxy of multimorbidity and not indicate causality, as reducing PIMs has not been shown to improve clinical outcomes such as hospitalization rates, duration of hospital stay, or total health care expenditure in patients with multimorbidity and polypharmacy [22,23]. In this context, it should be emphasized that the Beers Criteria list does not claim to enable prescribing decisions in clinical practice without further consideration of a person’s general health, medical conditions, laboratory results, or individual perceptions of the benefits and risks of pharmacotherapy. Nevertheless, even with the use of pharmavista’s filter for Beers alerts, resulting in 0.9 instead of 4.8 alerts per resident, the remaining alerts proved to be of limited help for clinical decision making and may just remind us that the critical evaluation of each drug’s indication and dose is an elemental part of best prescribing practice anyway. Besides the Beer Criteria list, there are also other collections of deprescribing guidelines (e.g., see https://deprescribing.org/deprescribing-guidelines-repository-depot/, access date 26 January 2025). However, detailed evaluations also concluded that these may provide little specific guidance on how recommendations should be implemented in clinical practice [24].

CNS drugs were of particular interest for our medication management, as they are well represented on the Beers Criteria list. Antidepressants, antipsychotics, benzodiazepines/Z-drugs, and opioids have a reputation for being overprescribed in the elderly, and CNS polypharmacy may be related to impaired cognitive function [25,26,27]. As shown in Table 2, the prevalence of CNS drug use was indeed high in our population. Nevertheless, our medication management did not result in an overall reduction.

Antipsychotics were usually well tolerated in our residents; particularly under quetiapine a relevant QTc prolongation is rare, and quetiapine is often prescribed as an alternative to benzodiazepines, which is a rational strategy and a common trend [28]. Similarly, antidepressants with sleep-inducing effects such as mirtazapine or trazodone can be prescribed as a generally well-tolerated alternative to benzodiazepines, and we assured that antidepressant users were monitored for hyponatremia and QTc prolongation.

For benzodiazepines/Z-drugs, the most common change we made was reducing the dose of zolpidem from 10 mg to the recommended 5 mg in older people. The reduction in and/or stopping of benzodiazepines was discussed with all users. However, a complete stop could mostly not be achieved, as many residents insisted that this would result in diminished sleep and quality of life, and some had already unsuccessfully tried this before. So, the reduction in benzodiazepine proved to be a delicate balance between the general aim to avoid their off-label long-term use vs. accepting the residents’ will and supporting their quality of life. Furthermore, it should also be mentioned that it remains unclear if the deprescribing of benzodiazepines/Z-drugs and other CNS drugs can effectively reduce falls in clinical practice [29,30].

Opioid treatment was also very common, but so was poorly controlled pain and its undertreatment in nursing home residents. Interestingly, polypharmacy, and therefore the aim to not further increase it, is a risk factor for the undertreatment of pain [31]. Therefore, we made sure that alternative treatment options including physiotherapy, paracetamol, metamizole, and NSAIDs were used as appropriate, but they were not able to reduce the overall use of opioids.

Other “classic” targets for deprescribing were apparently already well managed and did not require many changes in our population. As such, we rarely encountered inappropriate statin and aspirin use for the primary prevention of ischemic vascular disease in older people [32,33]; anticoagulant dose could be reduced in some residents but not stopped [34,35], and with the exception of metformin and metoclopramide, dose adjustments in response to renal impairment were also rarely necessary in spite of the 37.5% prevalence of renal impairment of stage 3 or more in our population.

Finally, we should mention some limitations of our study. We conducted an observational evaluation in a limited number of subjects without a control group, and residents therefore served as their own controls comparing baseline status vs. follow-up after proactive medication management. The quality of pharmacotherapy in the elderly may differ between institutions and health systems. However, we have a diverse team of internists, geriatricians, psychiatrists, and clinical pharmacologists who care for our nursing home residents, which is expected to equal out interpersonal variability in prescribing decisions, and other groups reported comparable prevalences for overall and drug-class-specific polypharmacy. We would therefore consider our results as being valid and also representable for other nursing homes. Also, we aimed to demonstrate the feasibility and present results of a local systematic real-life initiative for improved pharmacotherapy rather than a large controlled clinical trial that may not represent real-life conditions.

As mentioned above, we allowed for a long follow-up period to ensure that changes were persistent and well tolerated and did not need to be switched back to the original. The drawback of this long follow-up is that the residents’ physical condition may have further deteriorated and required an intensification of pharmacotherapy. However, Figure 3 allows at least a transparent interpretation of this possibility. Indeed, there was an increased use of analgesics, antidepressants, and antipsychotics, which is compatible with further deterioration, whereas medication classes that could be reduced overall stand out as they demonstrate a successful reduction in overmedication in older people.

Furthermore, it was beyond the scope and sample size of our study to measure the impact of proactive medication management on hospitalizations or potential cost savings. However, a study by Hurley et al. reported that deprescribing led to significant cost reductions in frail elderly nursing home residents [36], and we would therefore expect that our systematic check of each prescription and the stopping of at least one regular prescription in 64.3% of our nursing home residents likely led to reduced costs and improved clinical outcomes.

## 5. Conclusions

Local initiatives for proactive medication management in nursing home residents can increase awareness of the best prescribing practices in the elderly and lead to persistent medication changes in most residents, with the aim to improve efficacy and safety and reduce costs. On a larger scale, healthcare systems should incorporate systematic initiatives that evaluate and improve the quality of pharmacotherapy. However, a guideline- and algorithm-based reduction in overall polypharmacy may be neither realistic nor desirable, as nursing home residents require a personalized evaluation of their individual complex healthcare needs often in palliative situations. Therefore, individual initial and follow-up evaluations are required before and after medication changes. These may include the monitoring of drug-related outcomes such as blood pressure, sleep quality, or mood changes. In clinical practice as well as in future studies, improved quality of life should be the focus of proactive medication management and its subsequent evaluations.

## Figures and Tables

**Table 1:** Characteristics and baseline laboratory values of the study population.

	*n*	(%)
**Number of nursing home residents**	56	(100)
**Sex**		
Female	42	(75.0)
Male	14	(25.0)
**Age (years)**		
Median (range)	91	(74–100)
**Lead diagnosis**		
Polymorbidity	33	(58.9)
Dementia/cognitive impairment	19	(33.9)
Palliative care	3	(5.4)
Schizophrenia	1	(1.8)
Renal function (eGFR, mL/min/1.73 m^2^) *		
≥90	2	(4.2)
60–89	28	(58.3)
30–59	14	(29.2)
15–29	4	(8.3)
<15	0	(0)
Potassium *		
Hypokalaemia (<3.5 mmol/mL)	6	(10.7)
Hyperkalaemia (>5.0 mmol/mL)	0	(0)
Sodium *		
Hyponatraemia (<132 mmol/mL)	3	(5.4)
Hypernatraemia (>146 mmol/mL)	1	(1.8)

* Data available in the following proportions of subjects: eGFR 85.7%, K 80.4%, and Na 82.1%.

**Table 2:** Medication in 56 nursing home residents.

	Total	
	Mean	(Range)
Regular prescriptions per resident	7.9	(3–17)
On-demand prescriptions per resident	5.1	(0–13)
**Regular prescriptions**	*n* *	(%) *
Antihypertensives/diuretics	41	(73.2)
Anticoagulants/antiplatelets	30	(53.6)
Antiarrhythmics	25	(44.6)
Proton pump inhibitors	25	(44.6)
Antipsychotics	24	(42.9)
Antidepressants	21	(37.5)
Laxatives	22	(39.3)
Metamizole/paracetamol	20	(35.7)
Opioids	14	(25.0)
Cholesterol-lowering drugs	12	(21.4)
Benzodiazepines/Z-drugs	12	(21.4)
Antiepileptics (incl. pregabalin)	11	(19.6)
Glucose-lowering drugs	9	(16.1)
Hormones	9	(16.1)
Antiparkinson drugs	5	(8.9)
NSAIDs	2	(3.6)
Steroids and immunosuppressants	2	(3.6)
Antibiotics/antivirals/antifungals	1	(1.8)

* Number/percentage of patients with at least one prescription in each category.

**Table 3:** Frequency of CDSS alerts, stratified by category and potential severity.

	CDSS Alerts (Pharmavista)	
	Residents with ≥1 Alert (%)	*n* Alerts per Resident (Mean)
**Level 1 Alerts**	**20.0**	**0.3**
Drug–Age (Any)	0	0
Drug–Age (Beers) *	0	0
Drug–Drug Interaction	7.3	0.1
Renal Impairment	14.8	0.2
**Level 2 Alerts**	**90.9**	**5.7**
Drug–Age (Any)	50.9	0.7
Drug–Age (Beers) *	49.1	0.7
Drug–Drug Interaction	18.2	0.4
Renal Impairment	15.1	0.2
**Level 3 Alerts**	**89.1**	**6.7**
Drug–Age (Any)	3.6	0.2
Drug–Age (Beers) *	3.6	0.1
Drug–Drug Interaction	81.8	5.0
Renal Impairment	59.3	1.2

Level 1 = contraindicated: the two drugs must not be used together, with severe consequences likely. Level 2 = contraindicated as a precaution: the two drugs may not be taken together, with severe consequences being possible. Level 3 = monitoring/adaptation: alternative drugs, separate administration, dose adjustment, or monitoring recommended [12]. * with activation of pharmavista’s default filter for drug–age interactions.

## Data Availability

The data that support the findings of this study are available upon request from the corresponding author. The data are not publicly available due to privacy or ethical restrictions.
